# Supplementary material for: Identifying Inhibitors of −1 Programmed Ribosomal Frameshifting in a Broad Spectrum of Coronaviruses
Source: Viruses. 2022 Jan 18;14(2):177. doi: 10.3390/v14020177 (PMC8876150; doi:10.3390/v14020177)
Supplement: Supplementary file 1 [file viruses-14-00177-s001.zip › Munshi_etal_PanCoVFrameshiftInhibitors_SI.pdf]

Supplementary Material for:

Identifying inhibitors of −1 programmed ribosomal frameshifting in a broad spectrum of coronaviruses

Sneha Munshi,[1†] Krishna Neupane,[1†] Sandaru M. Ileperuma,[1] Matthew T.J. Halma,[1] Jamie A. Kelly,[2] Clarissa F. Halpern,[2] Jonathan D. Dinman,[2*] Sarah Loerch,[3‡*] Michael T. Woodside[1,4*]

[1]*Department of Physics, University of Alberta, Edmonton AB T6G 2E1 Canada*
[2]*Department of Cell Biology and Molecular Genetics, University of Maryland, College Park MD 20742 USA*
[3]*Janelia Research Campus, Howard Hughes Medical Institute, Ashburn VA 20147 USA*
[4]*Li Ka Shing Institute of Virology, University of Alberta, Edmonton AB T6G 2E1 Canada*
[‡]Current address: *Department of Chemistry and Biochemistry, University of California, Santa Cruz, Santa Cruz CA 95064 USA*

[†]These authors contributed equally
[*]Correspondence to: Jonathan Dinman (dinman@umd.edu), Sarah Loerch (sloerch@ucsc.edu), Michael Woodside (michael.woodside@ualberta.ca)

This PDF file includes:

Supplementary Tables 1–5
Supplementary Figures 1–5

Table S1: Frameshift signal sequences for panel used in testing inhibitors. Slippery sequence is underlined, spacer is shown in italic font.

Name	Sequence
KU182958	TTTTTAAAC*GAGTCA*GGGGTACTAGTGGAGTAGCCCGTCTAGTACCCCTAGGTTCTGGTG TAGAACCAGATATTGTATTAAGGGCTTT
LC469308	TTTTTAAAC*GAGTCC*GGGGTTCTATTGTAAATGCCCGAATAGAACCCTGTGCTAGTGGACT TTCCACTGATGTCGTTTTTAGGGCATT
KY770854	TTTTTAAAC*GAGTGC*GGGGCTCTAGTGATGCTCGACTAGAGCCCCTTAATGGCACTGACA CTGACAAGTGTGTTCGTGCTTTTGACGTTTACAACAAGGATGTTGCATGTATTGG
KF294282	TTTTTAAAC*GAGTGC*GGGGCTCTAGTGCAGCTCGACTAGAGCCCTGTAATGGTACTGAAC CTGAGCATGTGATTCGCGCCTT
MERS-CoV	TTTTTAAAC*GAGTCC*GGGGTTCTATTGTAAATGCCCGAATAGAACCCTGTTCAAGTGGTTT GTCCACTGATGTCGTCTTTAGGGCATT
SARS-CoV-2	TTTTTAAAC*GGGTTT*GCGGTGTAAGTGCAGCCCGTCTTACACCGTGCGGCACAGGCACT AGTACTGATGTCGTATACAGGGCTT
PEMV	TTTTTAAAC*GGGTAAT*TCCGGTCGACTCCGGAGAAACAAAGTCAATA
HIV	AATTTTTTA*GGGAAGAT*CTGGCCTTCCCACAAGGGAAGGCCAGGGAATTTTCTTC

Table S2: Hits from initial screen of drug library.

Hits altering only −1 PRF		Hits altering −1 PRF and general translation	
Febuxostat	Fostamatinib	Plerixafor	Azatadine
Tranilast	Erdafitinib	Idebenone	Ketoconazole
Bepridil	10-undecenoic acid	Ibudilast	Abemaciclib
Tafamidis	Santonin	Cariprazine	Ibrutinib
Brexpiprazol	Garenoxacin	Pasireotide	Maraviroc
Penbutolol	Cefmenoxime	Bremelanotide	Nelfinavir
Benzocaine	Tetracycline	Diclofenac	Ribostamycin
Omargliptin	Piromidic acid	Desloratadine	Streptomycin
Nafamostat	Dapsone		Azithromycin
Indinavir	Demeclocycline		
Nitazoxanide	Valnemulin		
Palbociclib	Raltitrexed		

Table S3: Oligonucleotides for bi-fluorescent reporter construction. Underlined bases in gBlock denote HIV-1 −1 PRF signal, lower-case bases in α-helix spacer denote linkers complementary to restriction sites for cloning.

Oligo name	Sequence
AcGFP1, mCherry, inteins, and HIV-1 −1 PRF gBlock	GCATCAGAGCAGATTGTACTGAGAGTGCACCATCTAGGGCGCGCCGGTACCGGTTCCAAG CTTGTTGACGCTAGCCTGCAGGCGGCCGCTAATACGACTCACTATAGGGCCACCATGGTG AGCAAGGGCGCCGAGCTGTTCACCGGCATCGTGCCCATCCTGATCGAGCTGAATGGCGA TGTGAATGGCCACAAGTTCAGCGTGAGCGGCGAGGGCGAGGGCGATGCCACCTACGGCA AGCTGACCCTGAAGTTCATCTGCACCACCGGCAAGCTGCCTGTGCCCTGGCCCACCCTGG TGACCACCCTGAGCTACGGCGTGCAGTGCTTCTCACGCTACCCCGATCACATGAAGCAGC ACGACTTCTTCAAGAGCGCCATGCCTGAGGGCTACATCCAGGAGCGCACCATCTTCTTCG AGGATGACGGCAACTACAAGTCGCGCGCCGAGGTGAAGTTCGAGGGCGATACCCTGGTG AATCGCATCGAGCTGACCGGCACCGATTTCAAGGAGGATGGCAACATCCTGGGCAATAAG ATGGAGTACAACTACAACGCCCACAATGTGTACATCATGACCGACAAGGCCAAGAATGGC ATCAAGGTGAACTTCAAGATCCGCCACAACATCGAGGATGGCAGCGTGCAGCTGGCCGAC

	CACTACCAGCAGAATACCCCCATCGGCGATGGCCCTGTGCTGCTGCCCGATAACCACTAC<br>CTGTCCACCCAGAGCGCCCTGTCCAAGGACCCCAACGAGAAGCGCGATCACATGATCTAC<br>TTCGGCTTCGTGACCGCCGCCGCCATCACCCACGGCATGGATGAGCTGTACAAGTATTTC<br>TACACCTCGAGAGAAGCCAGACACAAACAGAAAATTGTGGCACCGGTGAAACAGACTTTG<br>AATTTTGACCTTCTTAAGCTGGCGGGAGACGTCGAGTCCAACCCCGGGCCCtcGTCGACTT<br>TTTTAGGGAAGATCTGGCCTTCCCACAAGGGGAGGCCAGGGAATTTTCTTCCGAGCTCGA<br>AGACGCCAAAAACATAAAGAAAGGCCCGGCGCCATTCTATCCTCTAGAGGATGGAACCGC<br>TGGAGAGCAACTGCATAAGGCTGGATCCGAGGCACGGCATAAGCAAAAGATCGTAGCCCC<br>AGTAAAGCAAACACTCAACTTCGATCTACTCAAACTCGCAGGTGATGTGGAATCTAATCCA<br>GGACCTTTCGGATCTGCCACCATTATCGTGAGCAAGGGCGAGGAGGATAACATGGCCATC<br>ATCAAGGAGTTCATGCGCTTCAAGGTGCACATGGAGGGCTCCGTGAACGGCCACGAGTTC<br>GAGATCGAGGGCGAGGGCGAGGGCCGCCCCTACGAGGGCACCCAGACCGCCAAGCTGA<br>AGGTGACCAAGGGTGGCCCCCTGCCCTTCGCCTGGGACATCCTGTCCCCTCAGTTCATGT<br>ACGGCTCCAAGGCCTACGTGAAGCACCCCGCCGACATCCCCGACTACTTGAAGCTGTCCT<br>TCCCCGAGGGCTTCAAGTGGGAGCGCGTGATGAACTTCGAGGACGGCGGCGTGGTGACC<br>GTGACCCAGGACTCCTCCCTCCAGGACGGCGAGTTCATCTACAAGGTGAAGCTGCGCGG<br>CACCAACTTCCCCTCCGACGGCCCCGTAATGCAGAAGAAGACAATGGGCTGGGAGGCCT<br>CCTCCGAGCGGATGTACCCCGAGGACGGCGCCCTGAAGGGCGAGATCAAGCAGAGGCTG<br>AAGCTGAAGGACGGCGGCCACTACGACGCTGAGGTCAAGACCACCTACAAGGCCAAGAA<br>GCCCGTGCAGCTGCCCGGCGCCTACAACGTCAACATCAAGTTGGACATCACCTCCCACAA<br>CGAGGACTACACCATCGTGGAACAGTACGAACGCGCCGAGGGCCGCCACTCCACCGGCG<br>GCATGGACGAGCTGTACAAGTAGTTAATTAAAATAAAATATCTTTATTTTCATTACATCTGTG<br>TGTTGGTTTTTTGTGTGGTTTAGAGCGAGATTCCGTCTCAA
α-helix spacer top	tcgactcGAAGCAGCCGCTAAAGAGGCTGCGGCAAAAGCTg
α-helix spacer bottom	GATCCAGCTTTTGCCGCAGCCTCTTTAGCGGCTGCTTCGAG

Table S4: Library used for drug screening. Details of the drugs included in the screening library are available in the attached spreadsheet (TableS4.xlsx).

Table S5: Effects of −1 PRF inhibitors on general translation. The change in *Renilla* luciferase signal with drug present compared to that with drug absent was used to assay the effect of the inhibitors on general translation. Errors represent s.e.m. from 4 replicates.

**Compound**	**Change in *Renilla* luciferase signal (%)**
Nafamostat	−47 ± 9
Abemaciclib	−29 ± 5
Palbociclib	4 ± 15
Valnemulin	−28 ± 7

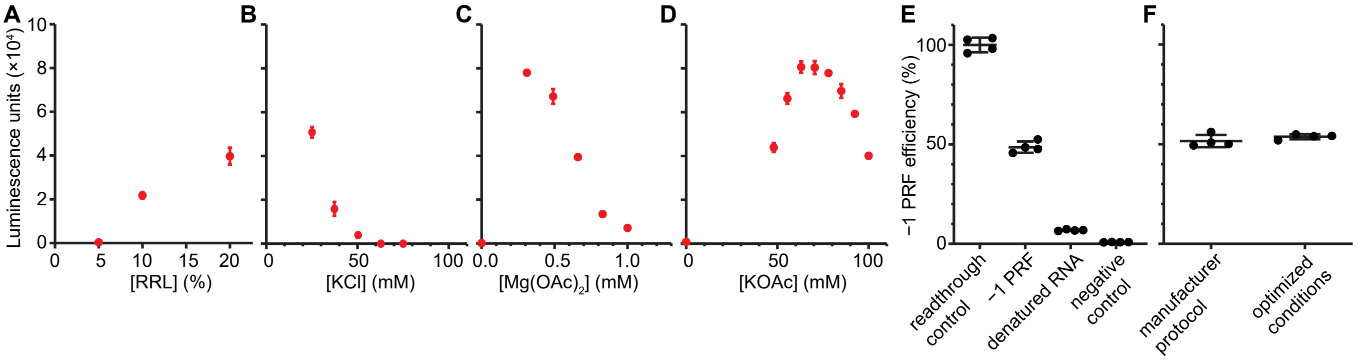

Figure S1: Optimization of dual-luciferase assay for high-throughput screening of −1 PRF in SARS-CoV-2. The following were sequentially optimized: (A) RRL content, (B) potassium chloride concentration, (C) magnesium acetate concentration, and (D) potassium acetate concentration. Each condition was measured in triplicate. (E) Using optimized conditions, ~50% −1 PRF efficiency was measured for the SARS-CoV-2 frameshift signal. −1 PRF was dependent on RNA structure: thermally denaturing RNA for 5 min at 95 °C followed by snap cooling on ice reduced −1 PRF efficiency to near zero. (F) Optimized conditions with 20% RRL have the same −1 PRF efficiency as reactions using the manufacturer protocol with 70% RRL (unpaired t-test with Welch's correction, p = 0.26).

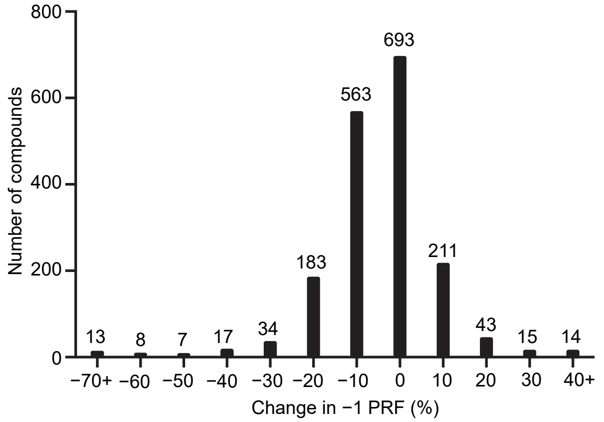

Figure S2: Results of drug-library screening. Distribution of effect sizes for change in −1 PRF using the SARS-CoV-2 frameshift reporter.

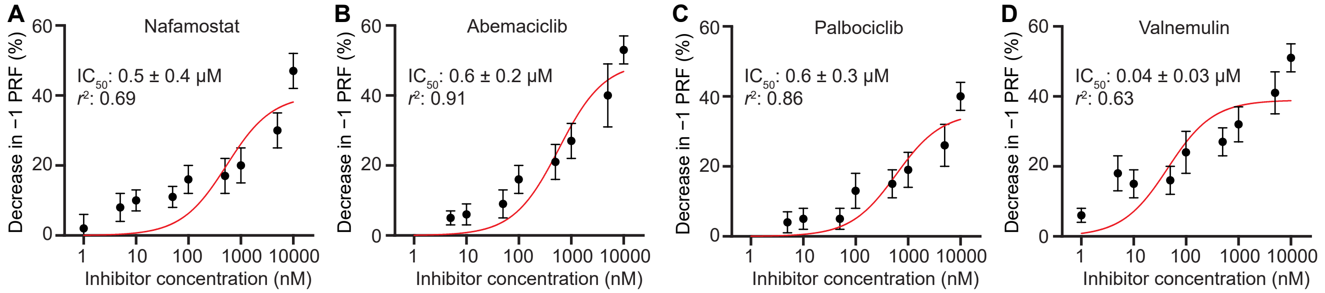

Figure S3: Dose-response curves for −1 PRF inhibition by compounds found in screen. Dose-response curves for (A) nafamostat, (B) abemaciclib, (C) palbociclib, and (D) valnemulin were fit to the Hill equation with slope 1 to find IC_{50}. Error bars represent standard error on the mean from 6 replicates.

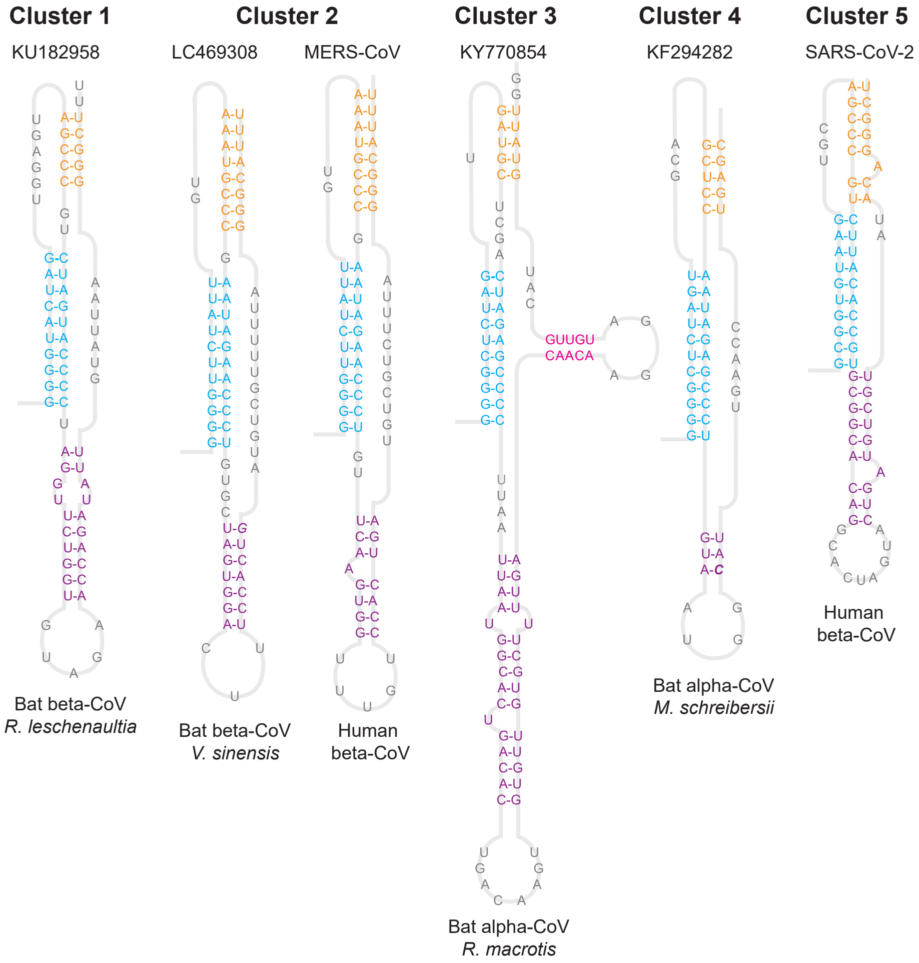

Figure S4: Secondary structure predictions for pseudoknots. Lowest-energy consensus structures homologous to the structures of the SARS-CoV and SARS-CoV-2 pseudoknots. Stem 1: cyan, stem 2: orange, stem 3: purple, stem 4: magenta, loops: grey.

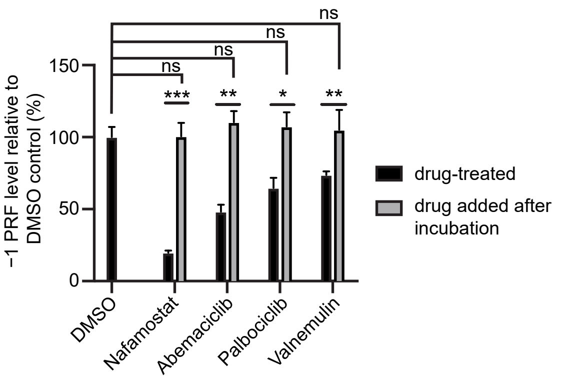

Figure S5: Testing for selective inhibition of firefly luciferase. The compounds from the drug screen that were used in testing against the panel of CoV frameshift signals did not selectively inhibit firefly luciferase: adding the drugs after incubating the translation product with luciferase assay reagents did not alter the firefly:*Renilla* luminescence ratio compared to the untreated case (unpaired t-test with Welch's correction, $p = 0.17$–0.95) and yielded a significantly different −1 PRF level compared to adding the drugs before translation ($p = 0.019$ to less than 0.0001).
